# Analysis and Characterization of Factors Affecting the Consistency of Tl-201 Myocardial Perfusion Single-Photon Emission Computed Tomography and Coronary Angiography Results in Patients with Suspected Coronary Artery Disease

**DOI:** 10.3390/diagnostics15121551

**Published:** 2025-06-18

**Authors:** Fu-Ren Tsai, Hung-Pin Chan, Chun-Hao Yin, Jin-Shuen Chen, Yao-Shen Chen, Daniel Hueng-Yuan Shen

**Affiliations:** 1 Department of Nuclear Medicine, Kaohsiung Veterans General Hospital, Kaohsiung 813, Taiwan; frtsai@vghks.gov.tw (F.-R.T.); markscience05@hotmail.com (H.-P.C.); 2 Department of Medical Education and Research, Kaohsiung Veterans General Hospital, Kaohsiung 813, Taiwan; chyin@vghks.gov.tw; 3 Institute of Health Care Management, National Sun Yat-sen University, Kaohsiung 804, Taiwan; 4 Department of Nursing, Meiho University, Pingtung 912, Taiwan; 5 Department of Administration, Kaohsiung Veterans General Hospital, Kaohsiung 813, Taiwan; dgschen@vghks.gov.tw (J.-S.C.); yschen@vghks.gov.tw (Y.-S.C.); 6 Department of Nuclear Medicine, Tri-Service General Hospital, Taipei 114, Taiwan

**Keywords:** myocardial perfusion imaging, diagnostic performance, patient characteristics

## Abstract

**Background/Objectives:** Tl-201 myocardial perfusion single-photon emission computed tomography (MPS) is a minimally invasive test for patients with suspected coronary artery disease (CAD). While its predictive and prognostic values are well established, diagnostic performance varies. A recent meta-analysis reported that the sensitivity and specificity of MPS range from 48.8 to 100% and 46.7 to 94.7%, respectively, reflecting discordance between CAG. Little is known, however, about the influence of patients’ characteristics and CAD risk factors on the diagnostic performance of MPS. This study aims to evaluate these factors in relation to MPS performance. **Methods**: We screened 4817 consecutive patients referred to our Nuclear Medicine Department in 2015 for Tl-201 MPS. Patients with clinically suspected ischemic heart disease who underwent CAG within 60 days post-MPS were included in the present analysis. The percentage of agreement/disagreement between the MPS-abnormal/normal and CAG-positive/negative groups was evaluated. Additionally, patient characteristics, CAD risk factors, co-morbidities, and single-photon emission computed tomography (SPECT) image-derived parameters were compared among the patients. **Results:** Among 635 patients with abnormal MPS, 583 had coronary stenosis. For the 52 without stenosis, causes included non-obstructive CAD (34.6%), prior infarction with scarring (32.7%), and imaging artifacts (32.7%). Significant stenosis was associated with older age, male sex, diabetes, dyslipidemia, CKD, and prior PCI, while hypertension and higher BMI were more common in insignificant CAD. Among 104 patients with normal MPS, 79 had stenosis, mainly in the LAD. Clinical risk factors were more prevalent in patients with any degree of stenosis. **Conclusions**: In patients with an abnormal MPS, the incorporation of visual interpretation, parameters, and CAD risk factors increases specificity and helps differentiate obstructive from non-obstructive CAD.

## 1. Introduction

Non-invasive assessment plays a pivotal role in coronary artery disease (CAD) by enabling early detection of atherosclerosis, guiding therapeutic decision-making, stratifying patient risk, and facilitating ongoing monitoring of disease progression or treatment response without the need for invasive procedures. While coronary CT angiography (CCTA) provides detailed anatomical visualization of coronary arteries to detect early-stage atheroma [1], myocardial perfusion single-photon emission computed tomography (MPS) remains irreplaceable because it uniquely assesses the functional significance of myocardial ischemia, offering physiological insight that anatomical imaging alone cannot provide. Although the predictive and prognostic value of myocardial perfusion SPECT (MPS) has been well established for decades, the diagnostic performance of MPS varies. According to a recent meta-analysis, when using conventional coronary angiography (CAG) as the gold standard for the diagnosis of CAD, the sensitivity and specificity of MPS range from 48.8 to 100% and 46.7 to 94.7%, respectively. These wide ranges indicate a significant amount of discordance between MPS and CAG. Several possible causes of false-positive and false-negative MPS results were mentioned in previous studies, including breast attenuation, diaphragmatic attenuation, overcorrection (i.e., sub-diaphragmatic activity), abnormal myocardial structure, and balanced ischemia. Little is known, however, about the influence of patients’ characteristics, CAD risk factors, and co-morbidities on the diagnostic performance of MPS. In the present study, therefore, we compared patient characteristics, CAD risk factors, and co-morbidities with the results of both concordant and discordant MPS scans.

## 2. Materials and Methods

Study Population: A total of 4817 adult patients who underwent Tl-201 dipyridamole-induced stress MPS tests at Kaohsiung Veterans General Hospital, Kaohsiung, Taiwan between January 2015 and December 2015 were retrospectively screened. Among the 4817 patients, 757 underwent CAG or percutaneous coronary intervention (PCI) within 60 days of the MPS, and the electronic medical records for those patients were then reviewed. After excluding 18 patients with missing or incomplete CAG data, a total of 739 patients were included in the medical record review and analysis.

Stress and Imaging Protocol: Patients were instructed to discontinue caffeinated foods, beverages, and xanthine-derived oral medications at least 24 h before testing. MPS was performed using a one-day stress-redistribution protocol as follows: dipyridamole was administered intravenously for 4 min at a dose of 0.56 mg/kg; thallium-201 was administered intravenously 7 min after the initiation of the dipyridamole injection; stress images were obtained, on average, 15 min after the thallium injection, and redistribution images were obtained 3 h later, both with patients in the prone position; single-photon emission computed tomography (SPECT) images were obtained in 64 projections over a 180° semicircular orbit (45° right anterior to 45° left posterior obliques) using a dual-headed gamma camera (Symbia Evo Excel, Siemens Healthineers, Erlangen, Germany) equipped with low-energy general-purpose collimators; image reconstruction was performed using filtered back projection with a Butterworth filter; raw cinematic and planar images (including short, horizontal long, and vertical long axes) were obtained, and a quantitative analysis using the Quantitative Perfusion SPECT (QPS) software version 2012 (Cedars-Sinai, Los Angeles, CA, USA) was performed.

Image Analysis and Interpretation: The SPECT images were interpreted by four nuclear medicine physicians with more than five years of experience each. Cinematic raw images of both stress and redistribution phases were evaluated by visual interpretation, and any incidental findings, such as increased lung uptake or left ventricle (LV) dilation, were documented. The location, severity, and reversibility of any perfusion defects were evaluated based on the reconstructed stress and rest SPECT images. The Quantitative Perfusion SPECT (QPS) application version 2012 (Cedars-Sinai, Los Angeles, CA, USA) was also utilized for quantitative assessment. QPS-derived parameters, including summed stress score (SSS), summed difference score (SDS), summed reversibility score (SRS), total perfusion deficit (TPD), and the extent of any perfusion defect during stress/redistribution, were documented. Diagnoses were made based on image findings and were categorized into two groups—“abnormal” and “normal”—based on the presence or absence, respectively, of a perfusion defect.

Coronary Angiography (CAG): All patients underwent elective CAG, performed by experienced cardiologists, within 60 days of the MPS. The degree of stenosis was assessed visually and documented, and patients were grouped based on the presence of hemodynamically significant CAD in any of the coronary vessels, which was defined as having ≥ 50% stenosis in the left main coronary artery (LM) or ≥70% stenosis in the left anterior descending artery (LAD), left circumflex artery (LCX), or right coronary artery (RCA). Findings other than atherosclerotic stenosis, including hypoplasia, small caliber, myocardial bridge, coronary spasm, and agenesis, were also documented. Patients were categorized into two groups based on the presence or absence of significant CAD.

Statistical Analysis: Patient demographic data were collected from the hospital’s health information system database. Risk factors and comorbidities were extracted using International Classification of Diseases, Tenth Revision (ICD-10) coding [2] and were presented as binary variables. Patients’ body weights and heights were extracted as numerical data. Patients were categorized into six groups based on the combination of MPS and CAG findings, and demographic data and parameters were compared among the groups. Statistical analyses were performed using IBM SPSS Statistics for Windows, Version 23.0 (IBM Corp., Armonk, NY, USA). A *t*-test was used to compare continuous numerical variables (i.e., age and body weight) among the groups, while the chi-squared test was used to compare binary variables.

## 3. Results

### 3.1. Patients

A total of 739 patients who met the inclusion criteria were enrolled in the study. Patients were aged from 33 to 101 years, with a median age of 66.5 years. More than two-thirds were male (n = 540, 73.1%). Approximately half of the patients had a history of PCI (n = 367, 49.7%), the majority of which were male (n = 299, 81.5%). The clinical characteristics of the patients are shown in Table 1.

### 3.2. MPS Findings

Of the 739 patients, 635 had an abnormal MPS, while 104 had normal results. The MPS-derived quantitative parameters of patients with abnormal and normal MPS results are shown in Table 2. Patients with an abnormal MPS had higher SSS, SRS, SDS, extent, and TPD (in both stress and redistribution images). LV dilation and increased lung uptake were observed more frequently in patients with abnormal MPS results. Additionally, all observed SPECT parameters differed significantly between patients with abnormal and normal MPS results.

### 3.3. CAG Findings

**Figure 1:**
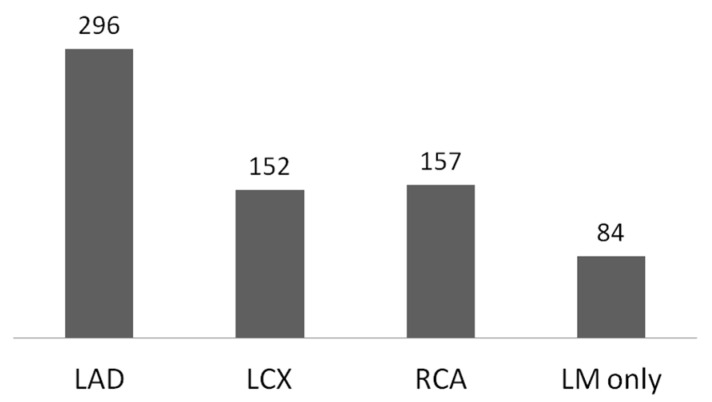
Coronary artery territory involved in patients with coronary artery disease.

Coronary stenosis was detected on CAG imaging in 662 patients, and the LAD was the most frequently involved coronary artery, followed by the RCA, LCX, and LM (Figure 1). Among the 662 patients with coronary stenosis, three-vessel disease was more frequent than two- or one-vessel disease. The angiographic findings of the coronary branches are shown in Table 3.

### 3.4. Agreement and Disagreement Between MPS and CAG

**Figure 2:**
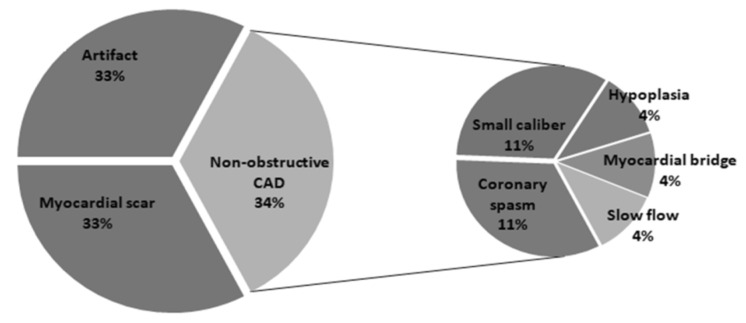
Coronary angiography (CAG) findings in patients with normal CAG but abnormal myocardial perfusion single-photon emission computed tomography (false positive).

Among the 635 patients with an abnormal MPS, 583 had coronary stenosis (473 significant and 110 insignificant). The most common branch involved was the TVD (35%), followed by the LAD (19.2%), LAD + LCX (11.5%), and LAD + RCA (11.3%) (Table 3). Among the 52 patients with no coronary stenosis on CAG imaging, 18 (34.6%) had non-obstructive CAD, 17 (32.7%) had history of myocardial infarction and were considered to have myocardial scarring, and 17 (32.7%) were determined to be imaging artifacts (Figure 2). Older age, male sex, diabetes, dyslipidemia, chronic kidney disease (CKD), and a history of previous PCI were most common in patients with significant coronary stenosis and second most common in those with insignificant coronary stenosis. On the contrary, body weight, body mass index, and rate of hypertension were most common in patients with insignificant CAD. MPS-derived parameters, including the prevalence of LV dilation, extent of any defect(s), TPD, SSS, SRS, and SDS, were higher in patients with significant coronary stenosis than in the other groups, but with no significant difference between patients with insignificant or no coronary stenosis.

Among the 104 patients with normal MPS results, 79 had coronary stenosis (36 significant and 43 insignificant), while only 25 had none. Among the 79 patients with coronary stenosis, the most common branch involved was the LAD (31.6%), followed by the LAD + LCX (17.7%) and LAD + RCA (15.2%) (Table 3). Older age was more common in patients with significant CAD than in those with insignificant or no CAD. Hypertension, diabetes, and dyslipidemia were more commonly observed in patients with significant or insignificant coronary stenosis than in those without. Other patient characteristics and SPECT parameters were not significantly different among the three groups.

## 4. Discussion

### 4.1. False Positives

In the present study, 52 of the 635 patients had abnormal MPS while no obstructive CAD was detected on CAG. Variations of the coronary arteries such as coronary spasm, myocardial bridge, small caliber coronary artery, and hypoplasia were observed in our patients with false-positive MPS results.

Coronary spasm has been reported in patients with chest pain syndrome and normal CAG [3]. Studies based on cardiac magnetic resonance imaging and quantitative myocardial perfusion imaging have revealed reduced coronary flow reserve in patients with coronary spasm, which may also cause reversible perfusion defects or reversed redistribution in MPS using Tl-201 or Tc-99m sestamibi [4,5,6,7]. Although PCI is a predictor of coronary spasm, coronary spasms are often associated with microvascular dysfunction [6] and multivessel coronary spasms are a major cause of cardiac arrest and sudden death [8].

The myocardial bridge is a congenital abnormality of the coronary artery where part of the epicardial coronary artery traverses intramuscularly within the myocardium and is compressed by the myocardium during systole. Because the maximal coronary blood supply occurs during the diastolic phase, compression of the coronary arteries during the systolic phase might not cause significant symptoms when the heart is not in an exertional condition. However, as the heart rate and contractility increase during exertional conditions, a shortened diastolic period and reduced coronary blood flow can result in a dissociation between blood supply and O2 demand, and this may eventually cause myocardial ischemia and sudden cardiac death [8]. MPI can provide assess for ischemia related to the myocardial bridge. Perfusion defects caused by myocardial bridges are seen not only in exercise stress MPS but also in pharmacological stress MPS [9,10,11].

Smaller diameter and hypoplastic coronary arteries are rare congenital coronary anomalies that account for hypoperfusion and sudden death in the absence of obstructive CAD [12]. These coronary anomalies are potential causes of false positives on CAG [13]. Perfusion defects caused by these coronary anomalies should not be considered as false positives since they do indicate an increased risk of cardiovascular events [12,13,14,15].

Imaging artifacts are also a common cause of false-positive or false-negative MPS results. Attenuation caused by the breast and diaphragm and the influence of strong subdiaphragmatic activity, motion, misalignment, myocardial creep, erroneous scaling, and segmentation are reported to be potential causes of false-positive and false-negative MPS results [16,17,18,19,20,21]. The adoption of techniques such as attenuation correction, prone or upright positioning, adequate rest after exercise stress, careful review of SPECT images, and the use of cinematic raw images may be helpful in increasing the accuracy of MPS.

### 4.2. False Negatives

The possible causes of false-negative MPS results have been previously discussed in the literature. Balanced ischemia [19,20] is a potential cause of false negatives in semiquantitative MPS, in which the detection of perfusion defects is based on the relative differences in perfusion between territories. The territory with the most abundant perfusion was regarded as normal, and territories with relatively abnormal perfusion were highlighted. If the perfusion decreased evenly across all territories with no significant differences among each other, the result of the MPS might appear normal. Patients with three-vessel disease (obstructive CAD involving the LAD, LCX and RCA, or LM and RCA) are often regarded as vulnerable to false-negative MPS results. Maaniitty et al. [22] utilized 15O-water positron emission tomography for the quantitative evaluation of myocardial blood flow, and a balanced reduction in myocardial blood flow was also observed in patients with no three-vessel disease. In the present study, false-negative MPS results were also observed in patients with single- and two-vessel disease.

A suboptimal stress test may also result in a false-negative MPS result. Stress test protocol states that in an exercise stress test, the endpoint of the exercise should not be merely based on heart rate but also on symptoms and changes in the electrocardiogram. In a pharmacological stress test, medications including caffeine, aminophylline, or beta-blockers that may interfere with the effect of vasodilators or dobutamine should be avoided [23].

### 4.3. Clinical Factors

Traditional CAD risk factors, including age, male sex, race, total cholesterol, HDL-C, blood pressure, blood pressure treatment status, diabetes, and current smoking status, were identified as strong predictors of 10-year CAD risk [24]. Patients with CKD have an increased risk of CAD due to exposure to other non-traditional uremia-related cardiovascular disease risk factors, such as inflammation, oxidative stress, and abnormal calcium-phosphorus metabolism. CKD is also considered an independent risk factor of CAD [25]. Age (male > 45 years, female > 55 years), sex, dyslipidemia (including abnormal total cholesterol, HDL-C, LDL-C, and TG levels), hypertension, smoking, and diabetes were obtained from the hospital information system database for further statistical analysis. Comorbidities, such as previous CAD and CKD, were also included in the present analysis, the results of which indicated that older age, male sex, diabetes, dyslipidemia, CKD, and a history of previous PCI were most common in patients with significant coronary stenosis, followed by insignificant coronary stenosis. Body weight, body mass index, and rate of hypertension, however, were most common in patients with insignificant CAD. In patients with abnormal MPS results, having three or more risk factors is a predictor of concordant coronary stenosis seen on CAG (sensitivity, 89%; specificity, 31%). In patients with normal CAG results, a higher number of risk factors is also associated with increased risk of coronary stenosis (sensitivity, 84%). Nakanishi et al. found that in patients with normal CAG results, pretest CAD probability > 66% was associated with high-risk CAD [26]. An increase in specificity (from 32.5% to 55.8%) of MPS was observed after incorporation of CAD risk factors into the interpretation of MPS images.

### 4.4. SPECT Parameters

The diagnostic and prognostic values of quantitative SPECT parameters have been described in many studies, indicating that perfusion abnormality involving >5% of the myocardium is predictive of increased risk of cardiac death. A segment-based summed score incorporating the extent and severity of perfusion has been regarded as a better predictive method and has been widely applied in other studies [27]. An SSS < 4 was associated with <1% per year incidence of severe cardiovascular events, such as cardiac death or nonfatal myocardial infarction. For patients with an SSS > 8, the incidence of severe cardiovascular events increased to 3.9% per year [28]. In patients with abnormal MPS results, LV dilation and increased quantitative parameters such as the extent of any perfusion defects, TPD, and SSS were associated with both significant and insignificant coronary stenosis. In the present study, an SSS of eight seemed to be the most preferred cutoff, yielding the best accuracy in the diagnosis of significant CAD (area under the curve, 0.644). A wide range of variations in the quantitative parameters was also observed in the present study. The variability in these quantitative parameters may originate from differences in the sex, body habitus, and soft tissue attenuation of the patients [29], which is a limitation of the present study.

### 4.5. Limitations

This study has several limitations. First, the interpretation of myocardial perfusion single-photon emission computed tomography (MPS) and coronary angiography (CAG) was based on local visual assessment rather than core laboratory adjudication, which may introduce inter-observer variability and diagnostic subjectivity. Second, quantitative coronary angiography (QCA) was not utilized, thereby limiting the precision and reproducibility of stenosis measurements. Third, visual estimation is prone to the oculostenotic reflex—a well-documented bias in which intermediate lesions are often overestimated in severity without objective evidence of functional significance. Fourth, MPS findings were not validated using pressure wire–based physiological assessments such as fractional flow reserve (FFR) or instantaneous wave-free ratio (iFR), both of which are considered contemporary gold standards for evaluating lesion-specific ischemia. Lastly, the absence of standardized physiologic reference techniques in the assessment of coronary stenosis may compromise the accuracy of comparisons and the interpretation of MPS results.

## 5. Conclusions

In patients with suspected CAD who have both an abnormal MPS result and traditional risk factors, such as hypertension, diabetes, dyslipidemia, and CKD, are associated with greater likelihood of concordant CAG results of significant CAD.

## Figures and Tables

**Table 1:** Patient characteristics.

Parameters	Number (n = 739)	Percentage (%)
Age	66.4 ± 12.2	
Gender		
Male	540	73.1
Female	199	26.9
Body height	163.0 ± 9.1	
Body weight	70.1 ± 13.3	
Body mass index	26.6 ± 9.1	
Smoking	366	49.5
Alcohol consumption	243	32.9
Hypertension	582	78.8
Diabetes	339	45.9
Dyslipidemia	430	58.2
Hemodialysis	114	15.4
Previous percutaneous coronary intervention ^1^	367	49.7

^1^ History of percutaneous coronary intervention (PCI). Age, body height, body weight, and body mass index were presented as mean ± SD.

**Table 2:** Myocardial perfusion single-photon emission computed tomography parameters.

	Visual Interpretation		
	Abnormal (n = 635)	Normal (n = 104)	*p*-Value
Increased lung uptake	476 (75.0%)	65 (62.5%)	0.008
Left ventricular dilatation	387 (60.9%)	21 (20.2%)	<0.001
Stress extent	15.3 ± 14.7	1.9 ± 4.2	<0.001
Redistributed extent	8.8 ± 11.8	2.3 ± 4.1	<0.001
Stress total perfusion deficit	12.4 ± 11.5	2.1 ± 3.3	<0.001
Redistributed total perfusion deficit	7.5 ± 9.4	2.3 ± 3.2	<0.001
Summed stress score	9.2 ± 8.4	1.4 ± 2.8	<0.001
Summed reversibility score	4.6 ± 6.4	0.6 ± 2.1	<0.001
Summed difference score	4.3 ± 4.8	0.8 ± 1.1	<0.001

**Table 3:** Coronary angiography findings in patients with significant or insignificant coronary stenosis.

	Abnormal MPS (n = 583)	Normal MPS (n = 79)	Total (n = 662)
Single vessel disease			
LAD	112 (19.2%)	25 (31.6%)	137 (20.7%)
LCX	31 (5.3%)	10 (12.7%)	41 (6.2%)
RCA	43 (7.4%)	6 (7.6%)	49 (7.4%)
Double vessel disease			
LAD + LCX	67 (11.5%)	14 (17.7%)	81 (12.2%)
LAD + RCA	66 (11.3%)	12 (15.2%)	78 (11.8%)
LCX + RCA	27 (4.6%)	3 (3.8%)	30 (4.5%)
Three-vessel disease	204 (35.0%)	7 (8.9%)	211 (31.9%)
LM only	33 (5.7%)	2 (2.5%)	35 (5.3%)

LAD, left anterior descending artery; LCX, left circumflex artery; RCA, right coronary artery.

## Data Availability

The data presented in this study are available on request from the corresponding author. The data are not publicly available due to privacy considerations.

## Abbreviations

The following abbreviations are used in this manuscript:

CAD	Coronary artery disease
CAG	Coronary angiography
LAD	Left anterior descending artery
LCX	Left circumflex artery
LM	Left main coronary artery
MPS	Myocardial perfusion single-photon emission computed tomography
PCI	Percutaneous coronary intervention
QPS	Quantitative perfusion single-photon emission computed tomography
RCA	Right coronary artery
SPECT	Single-photon emission computed tomography
SSS	Summed stress score
SDS	Summed difference score
SRS	Summed reversibility score
TPD	Total perfusion deficit

## References

[1] Zaman S., Wasfy J.H., Kapil V., Ziaeian B., Parsonage W.A., Sriswasdi S., Chico T.J.A., Capodanno D., Colleran R., Sutton N.R. (2025). The Lancet Commission on rethinking coronary artery disease: Moving from ischaemia to atheroma. Lancet.

[2] World Health Organization (2019). International Statistical Classification of Diseases and Related Health Problems, 10th Revision (ICD-10).

[3] Jenkins K., Pompei G., Ganzorig N., Brown S., Beltrame J., Kunadian V. (2024). Vasospastic angina: A review on diagnostic approach and management. Ther. Adv. Cardiovasc. Dis..

[4] Pirozzolo G., Martinez Pereyra V., Hubert A., Guenther F., Sechtem U., Bekeredjian R., Mahrholdt H., Ong P., Seitz A. (2021). Coronary artery spasm and impaired myocardial perfusion in patients with ANOCA: Predictors from a multimodality study using stress CMR and acetylcholine testing. Int. J. Cardiol..

[5] Chen Y., Pang Z.K., Wang J., Yang R.F., Jing R., Chu H.X., Hsu B., Lin W.H., Li J.M. (2022). Serial Changes of (99m)Tc-Sestamibi Washout Due to Coronary Spasm Captured by Dynamic Myocardial Perfusion Imaging With Cardiac Dedicated CZT-SPECT: A Case Report. Circ. Cardiovasc. Imaging.

[6] Abramik J., Mariathas M., Felekos I. (2025). Coronary Microvascular Dysfunction and Vasospastic Angina—Pathophysiology, Diagnosis and Management Strategies. J. Clin. Med..

[7] Vink C.E.M., Borodzicz-Jazdzyk S., de Jong E.A.M., Woudstra J., van de Hoef T.P., Chamuleau S.A.J., Eringa E.C., Götte M.J.W., Appelman Y. (2025). Quantitative perfusion by cardiac magnetic resonance imaging reveals compromised myocardial perfusion in patients with angina with non-obstructive coronary artery disease. Clin. Res. Cardiol..

[8] Favorini S., Perrin T., Hellige G., Arenja N. (2023). Sudden cardiac arrest due to recurrent coronary spasm in a young woman: A case report. Eur. Heart J. Case Rep..

[9] Oh S., Hyun D.Y., Cho S.-G., Hong Y.J., Kim J.H., Ahn Y., Jeong M.H. (2023). Case report: A fatal case of myocardial infarction due to myocardial bridge and concomitant vasospasm: The role of stress gated SPECT. Front. Cardiovasc. Med..

[10] Ker W.D.S., Neves D.G., Damas A., Mesquita C.T., Nacif M.S. (2017). Myocardial Bridge and Angiotomography of the Coronary Arteries: Perfusion under Pharmacological Stress. Arq. Bras. Cardiol..

[11] Sternheim D., Power D.A., Samtani R., Kini A., Fuster V., Sharma S. (2021). Myocardial Bridging: Diagnosis, Functional Assessment, and Management: JACC State-of-the-Art Review. J. Am. Coll. Cardiol..

[12] Zamfir A.-S., Stătescu C., Sascău R.A., Tinică G., Zamfir C.L., Cernomaz T.-A., Chistol R.O., Boișteanu D., Sava A. (2024). Casting Light on The Hidden Prevalence: A Novel Perspective on Hypoplastic Coronary Artery Disease. J. Clin. Med..

[13] Guo A., Bakhshi H., O’Hara J., Genovese L., Fein A., Maghsoudi A., Sandesara C. (2021). Hypoplastic Coronary Artery Disease Presenting with Ventricular Fibrillation Cardiac Arrest. Eur. J. Case Rep. Intern. Med..

[14] De Giorgio F., Abbate A., Stigliano E., Capelli A., Arena V. (2010). Hypoplastic coronary artery disease causing sudden death. Report of two cases and review of the literature. Cardiovasc. Pathol..

[15] Villa A.D., Sammut E., Nair A., Rajani R., Bonamini R., Chiribiri A. (2016). Coronary artery anomalies overview: The normal and the abnormal. World J. Radiol..

[16] Johnson S.G. (2025). Motion Artifacts in SPECT Myocardial Perfusion Imaging. J. Nucl. Med. Technol..

[17] Malek H., Yaghoobi N., Hedayati R. (2017). Artifacts in Quantitative analysis of myocardial perfusion SPECT, using Cedars-Sinai QPS Software. J. Nucl. Cardiol..

[18] Ramos S.M.O., Glavam A.P., de Brito A.S.X., Kubo T.T.A., Tukamoto G., Sampaio D., de Sá L.V. (2020). Prone Myocardial Perfusion Imaging and Breast Attenuation: A Phantom Study. Curr. Med. Imaging Rev..

[19] Strok A., Salobir B.G., Stalc M., Zaletel K. (2024). Subdiaphragmatic activity-related artifacts in myocardial perfusion scintigraphy. Radiol. Oncol..

[20] Tawakol A.E., Tantawy H.M., Elashmawy R.E., Abdelhafez Y.G., Elsayed Y.M. (2021). Added Value of CT Attenuation Correction and Prone Positioning in Improving Breast and Subdiaphragmatic Attenuation in Myocardial Perfusion Imaging. J. Nucl. Med. Technol..

[21] von Felten E., Benetos G., Patriki D., Benz D.C., Rampidis G.P., Giannopoulos A.A., Bakula A., Gräni C., Pazhenkottil A.P., Gebhard C. (2021). Myocardial creep-induced misalignment artifacts in PET/MR myocardial perfusion imaging. Eur. J. Nucl. Med. Mol. Imaging.

[22] Maaniitty T., Stenstrom I., Saraste A., Knuuti J. (2021). Extensive and balanced reduction of myocardial blood flow in patients with suspected obstructive coronary artery disease: 15O-water PET study. Int. J. Cardiol..

[23] Mann A., Williams J. (2020). Considerations for Stress Testing Performed in Conjunction with Myocardial Perfusion Imaging. J. Nucl. Med. Technol..

[24] Hajar R. (2017). Risk Factors for Coronary Artery Disease: Historical Perspectives. Heart Views.

[25] Sarnak M.J., Amann K., Bangalore S., Cavalcante J.L., Charytan D.M., Craig J.C., Gill J.S., Hlatky M.A., Jardine A.G., Landmesser U. (2019). Chronic Kidney Disease and Coronary Artery Disease: JACC State-of-the-Art Review. J. Am. Coll. Cardiol..

[26] Nakanishi R., Gransar H., Slomka P., Arsanjani R., Shalev A., Otaki Y., Friedman J.D., Hayes S.W., Thomson L.E., Fish M. (2016). Predictors of high-risk coronary artery disease in subjects with normal SPECT myocardial perfusion imaging. J. Nucl. Cardiol..

[27] Berman D.S., Abidov A., Kang X., Hayes S.W., Friedman J.D., Sciammarella M.G., Cohen I., Gerlach J., Waechter P.B., Germano G. (2004). Prognostic validation of a 17-segment score derived from a 20-segment score for myocardial perfusion SPECT interpretation. J. Nucl. Cardiol..

[28] Hachamovitch R., Berman D.S., Kiat H., Cohen I., Friedman J.D., Shaw L.J. (2002). Value of Stress Myocardial Perfusion Single Photon Emission Computed Tomography in Patients With Normal Resting Electrocardiograms. Circulation.

[29] DePuey E.G. (2016). Sources of variability of gated myocardial perfusion SPECT quantitative parameters. J. Nucl. Cardiol..

